# Treatment of Meatal Strictures by Dorsal Inlay Oral Mucosa Graft Urethroplasty: A Single-Center Experience

**DOI:** 10.3390/jcm10194312

**Published:** 2021-09-22

**Authors:** Michel Wirtz, Wietse Claeys, Philippe Francois, Marjan Waterloos, Mieke Waterschoot, Nicolaas Lumen

**Affiliations:** 1 Department of Urology, Ghent University Hospital, 9000 Gent, Belgium; wietse.claeys@uzgent.be (W.C.); marjan.waterloos@uzgent.be (M.W.); mieke.waterschoot@uzgent.be (M.W.); nicolaas.lumen@uzgent.be (N.L.); 2 Centre Hospitalier de Mouscron, 7700 Mouscron, Belgium; p.francois@chmouscron.be

**Keywords:** urethra, urethral stricture, meatus, meatal stricture, meatal stenosis, oral mucosa, urethroplasty

## Abstract

Background: To report on the use of oral mucosa graft urethroplasty for meatal strictures using the dorsal inlay technique. Methods: Patients who underwent a single-stage dorsal inlay oral mucosal graft urethroplasty between January 2000 and May 2021 were included in this study. A follow-up of a minimum of 12 months was necessary for inclusion. Exclusion criteria were stricture extension into the penile urethra, concomitant stricture at another location, flap urethroplasty for a meatal stricture, dorsal inlay urethroplasty with another type of graft, ventral onlay graft urethroplasty or staged urethroplasty. Recurrence was defined by the inability to pass a 14F metal sound through the reconstructed meatus irrespective of patients’ complaints. Results: Our study cohort included 40 patients. Buccal mucosal graft (BMG) urethroplasty was used in 25 patients and 15 patients were treated with the aid of lingual mucosal graft (LMG). The median follow-up was 85 (IQR: 69–110) months. Seven (17.5%) patients suffered a stricture recurrence of which four (10%) needed re-intervention. The median 5-y recurrent free survival (RFS) for the entire cohort was 85 (±6)%. The median 5-y RFS was 96 (±4)% versus 65 (±13)% for respectively BMG and LMG (*p* = 0.03). Post-operative complications were identified in 11 (27.5%) patients with only one (2.5%) patient who had a grade 3a complication. Conclusions: Dorsal inlay oral mucosa graft urethroplasty is a safe and feasible technique for selected patients with meatal stenosis.

## 1. Introduction

The etiology of a meatal stricture typically includes iatrogenic factors (such as failed hypospadias repair, urethral instrumentation), genital lichen sclerosus (LS) and external trauma [1,2]. Meatal strictures are a challenging problem for reconstructive urologists.

First-line treatment might be dilatation but this is rarely curative. Urethroplasty offers a more durable chance of long-term urethral patency. Several techniques of urethroplasty for meatal strictures have been described but at present, graft augmentation urethroplasty in a single-stage or staged approach is one of the standard options [3]. Because of the high chance of (concomitant) LS as etiology for a meatal stricture, the use of genital skin as a substitute should be avoided because LS can further affect this substitute with the risk of stricture recurrence [3]. Oral mucosa grafts are more resilient to LS and are preferred as a graft at the meatus [4,5].

For meatal strictures and penile strictures in general a ventral onlay approach is possible but yields inferior outcomes [6]. This is mainly due to the anatomy of the penile urethra which has thinner ventral spongious tissue in contrast to the bulbar urethra [7]. The consequence of this is less vascular support from the tissues covering the graft and therefore, a greater risk of ballooning and urine pooling, which affects graft survival and increases the risk of stricture recurrence. Consequently, dorsal grafting techniques have been developed [7,8]. At the meatus, a dorsal [9,10] or dorsolateral onlay approach [8] is not possible. However, the Asopa’s technique consisting of a dorsal inlay grafting through a ventral sagittal approach is a potential dorsal grafting technique [11]. At the meatus, the abundant dorsal spongious tissue of the glans usually provides a suitable graft bed.

The aim of this study was to report on the use of oral mucosa graft urethroplasty using Asopa’s technique in a cohort of patients suffering from a meatal stricture. Furthermore, we wanted to explore whether there were any differences in outcomes between LS, failed hypospadias repair (FHR) and other etiologies and between buccal and lingual mucosa as graft substitutes.

## 2. Materials and Methods

### 2.1. Patient Population

Since 2000, patients undergoing urethroplasty for urethral stricture disease at Ghent University Hospital were enrolled in a database after having obtained their informed consent (Ethics committee approval EC/UZG 2008/234). This database collects information about all known relevant patient and stricture characteristics. At data cut-off (14 May 2021), this database contained over 1200 cases. A search was performed to select patients who underwent oral mucosa graft dorsal inlay urethroplasty for meatal strictures. Patients were included if a minimum follow-up of 12 months was available. Patients were excluded if they had stricture extension into the penile urethra, concomitant stricture at another location, flap urethroplasty for a meatal stricture, dorsal inlay urethroplasty with another type of graft, ventral onlay graft urethroplasty or staged urethroplasty. The preoperative evaluation of the patients includes history taking, uroflowmetry and echographic residual volume measurement, clinical examination, urine culture and urethrography. The potential etiology of the stricture was carefully questioned. Both LS and FHR-related strictures are a specific etiology for meatal strictures and therefore we decided to group patients into “LS and no-LS etiology” and into “FHR and no-FHR etiology”. After surgery, the patients were followed after 3 months, 12 months and annually thereafter. Post-operative visits included history taking, clinical evaluation, calibration (14F) of the meatus, uroflowmetry and echographic residual measurement. Stricture recurrence was defined as the inability to pass a 14F metal sound through the reconstructed meatus (irrespective of whether or not the patients’ mentioned complaints or needed further surgery, i.e., anatomical definition of recurrence). Complications within 3 months were classified according to Clavien-Dindo classification.

### 2.2. Surgical Technique

All patients were treated by general anesthesia and a single shot of 2 g of cefazolin was given at induction in patients with a negative urine culture. Patients with a urinary tract infection were treated with antibiotics before the operation. Patients with a suprapubic catheter started with antibiotics according to antibiogram the day before surgery. Patients were treated in the common supine position. A stay suture was placed at the tip of the glans. A grooved metal sound or a 3F ureteric catheter was introduced through the meatus as a guidewire. A tourniquet was applied at the base of the penis. The glans and urethra were incised ventrally. The stricture was further opened along the guide until healthy urethra was encountered that allowed easy passage of a 20F metal sound. Stay sutures were placed at the urethral mucosa and this mucosa was colored by blue ink. The urethral plate was then incised dorsally deep into the glandular tissue. By doing so, a gap of spongious tissue was created between the urethral edges. Severe spongiofibrosis was resected until a healthy vascular bed was created to ensure graft ingrowth. The resected tissue was sent for pathological examination. A shield-shaped graft was harvested either from the inner surface of the cheek (buccal mucosa) or dorsolateral surface of the tongue (lingual mucosa) according to the dimension of the gap that was created. The type of graft was dictated by the preference of the surgeon or patient or by previous oral harvesting (e.g., lingual mucosa in case of previous buccal mucosa and vice versa). The graft was defatted and meshed. The graft was then transferred to the gap in the urethral plate and the edges of the grafts were sutured to the urethral edges using monocryl™ 4.0. Dorsal quilting was performed to ensure close contact between the graft and the underlying graft bed. The urethra was closed ventrally using running suture monocryl™ 4.0 after the insertion of a 20F urethral catheter. Glans wings were created by a bilateral deep incision in the glans on both sides of the urethra. These glans wings were approximated ventrally over the urethra. The more proximal part of the closed urethra was covered with the remaining dartos tissue. The skin was closed with Vicryl Rapide™ 4.0. The patients were usually discharged the day after surgery when they resumed oral food intake. The catheter was maintained for 14 days and was only removed if the peri-catheter instillation and/or urethrography showed no significant extravasation. In the case of extravasation, the catheter was maintained for an additional week until no further extravasation was noted.

### 2.3. Statistical Analysis

Because of the small sample size, all test were non-parametric. Descriptive statistics were used to characterize the study population. Among subgroups, categorical and continuous data were compared using respectively Fisher’s exact test and the Mann-Whitney U test. Recurrence-free survival (RFS) was calculated using Kaplan-Meier statistics and the subgroups were compared using the log-rank test.

## 3. Results

At present, the database includes information about 1205 urethroplasties. The Asopa’s technique was used at the meatus in 63 cases. Respectively 12 and 11 cases were further excluded because of the use of a preputial skin graft and no pure meatal stricture location. This finally yielded 40 patients which represent the current study cohort. Stricture etiology was LS, FHR, transurethral resection, external trauma, inflammatory and idiopathic in respectively 18, 8, 2, 1, 1 and 10 cases. Respectively 25 and 15 patients were treated with the aid of a buccal mucosal graft (BMG) and lingual mucosal graft (LMG). The mean preoperative maximum flow rate on the uroflowmetry was 8.36 (SD: 4.64) mL/s. The average flow rate was 6.03 (SD: 3.43) mL/s; both of these for a mean voided volume of 228 (SD: 187) mL. The preoperative post-void residual volume was not clinically significant.

At the time of surgery, four (10%) patients had a suprapubic catheter to ensure urinary drainage. Patient and stricture characteristics are further summarized in Table 1. Notably, patients treated with LMG were more treatment naive as compared to those treated with BMG (resp. 53% versus 12%; *p* = 0.006). Patients with FHR as etiology were younger compared those suffering from other etiologies (resp. 35 versus 49 years; *p* = 0.046). All other characteristics were comparable across subgroups.

With a median follow-up of 85 (IQR: 69–110) months, seven (17.5%) patients suffered a stricture recurrence of which four (10%) needed re-intervention and three (7.5%) were followed without intervention because they were satisfied with the result. The median 5-y RFS for the entire cohort was 85 (±6)%. The median 5-y RFS was 96 (±4)% versus 65 (±13)% for respectively the BMG and LMG (*p* = 0.03), 89 (±7)% versus 81 (±9)% for respectively the LS and no-LS (*p* = 0.85) and 63 (±17)% versus 90 (±5)% for respectively the FHR versus the no-FHR (*p* = 0.08).

Post-operative complications were identified in eleven (27.5%) patients: six (15%), four (10%) and one (2.5%) patients had respectively a grade 1, 2 and 3a complication. Minor complications included temporary fistula (*n* = 2), wound infection (*n* = 3), urinary tract infection (*n* = 1), wound dehiscence (*n* = 1) and temporary penile edema (*n* = 3). The patient with a major complication suffered urinary retention requiring the insertion of a suprapubic catheter under local anesthetics. There were no significant differences in complications across subgroups. The median hospital stay was 2 (IQR: 1–2) days and the median operation time was 80 (IQR: 65–90) min. Of note, the operation time for LS was shorter compared to other non-LS related strictures (resp. 71 versus 85 min; *p* = 0.048). The first postoperative maximum and average flow rates were significantly better (resp. 8.36 mL/s versus 21.34 mL/s, *p* < 0.001). The data were too sparsely scattered to prove any significant differences in voiding function between the pre-defined subgroups. No data were recorded regarding erectile dysfunction in the pre- and post-operative setting. Further details of the outcome of Asopa’s technique are detailed in Table 2 and voiding outcomes in Table 3.

## 4. Discussion

Distal urethral strictures include meatal and glandular stenosis and are due to trauma, infections, iatrogenic instrumentalizations, skin diseases or can occur idiopathically. They represent a non-negligible sub-group of urethral stenosis. Besides dilatations and urethrotomies, which yield non satisfactory results, surgical intervention, such as single or staged augmentation urethroplasties with transplantation of autogenous genital or non-genital tissue, may be performed.

Isolated meatal strictures can be treated with different operative techniques. Firstly, the Malone technique, described by Peter Malone in 2004 [12], yields good functional and aesthetical results, but can only be used for short strictures of the external urinary meatus. In our cohort study the median urethral stricture length is 2 cm and therefore too long to apply this technique.

Another surgical option for meatal stenosis including the distal part of the urethra is the staged urethroplasty, which was first described by Bengt Johanson [13] in 1953. Although this staged approach is always possible, it means needing at least two operations with 3 to 6 months between them for a good healing of the dorsal plate with a non-aesthetic aspect of the glans and penis in the meantime.

In 1968 a new surgical one-stage urethroplasty technique was presented by Orandi [14] for the repair of urethral strictures. It consists of a one-stage flap urethroplasty using the principles of pedicled skin grafting. After that, free full thickness skin grafts have been utilized in the treatment of urethral strictures [15]. Preputial skin or post-auricular skin can be used, but in the case of lichen sclerosus they are not indicated because the grafts risk to develop the skin disease as well [16,17].

In 2016, Nikolavsky et al. [18] presented a new surgical single-stage technique and their preliminary results using a transurethral ventral BMG inlay urethroplasty. In this case report of three patients the reconstruction of the distal urethra was conducted using a buccal mucosal graft through a transurethral stricture excision without skin incision or urethral mobilization. They hypothesized that this technique yields good results without glans dehiscence and fistula formation. In 2020, an international multi-institutional retrospective study by Daneshvar et al. [19] reported on this same technique. They included 68 patients with a median stricture length of 2 cm. They conclude that the transurethral approach has a great aesthetic advantage not needing any skin incisions and seems to have a very good functional outcome and patency rate (95% remaining stricture-free at a limited median follow-up of only 17 months). We hypothesize on the other hand that the learning curve of this technique is higher given the nearly blind stricture excision and graft positioning. This seems even more obvious in LS strictures where a pin-hole meatus might be present as well. Therefore, it might occur that the defect of the excised stricture is not completely covered distally, leaving a gap susceptible for inappropriate healing and stricture formation. Furthermore, it seems that this technique does not result in a slid-like meatus, yielding in a possible risk of postoperative spray stream even though the authors stated no increase in urinary spraying.

Other surgical techniques, such as a dorsal [9,10] or dorso-lateral [8] approach for longer strictures at the meatus are not possible due to its anatomy.

To our knowledge, the use of the Asopa technique, a dorsal inlay grafting through a ventral sagittal approach in distal penile strictures has only been described in a few articles [20,21,22,23,24], of which only two publications described this technique for isolated meatal strictures [21,25]. Zumstein et al. [20] did a retrospective analysis of 125 patients who underwent a single-stage Asopa urethroplasty for penile strictures. Thirty-eight patients (30%) had a distal penile stenosis. With a median follow-up of 36 months, the RFS rate for distal penile strictures was 66%. They found that the patients with distal penile structures were younger and that the graft length was significantly shorter compared to other stricture locations. Unfortunately, they excluded patients with isolated meatal or fossa navicularis strictures. Pisapati et al. [22] prospectively analyzed their results of dorsal inlay buccal mucosa graft urethroplasty. Their overall success rate was 87%, but the distal urethra was only involved in eight of their forty-five cases. The etiology of strictures was reported to be lichen sclerosus in seventeen cases.

In 2008 Barbagli et al. [21] published a retrospective analysis on 63 patients who were treated with a one-stage penile urethroplasty using a flap or a graft for reconstruction of the urethra. In 26 (41%) patients the stricture involved the distal penile urethra and the fossa navicularis, including the external urinary meatus. Nineteen patients were treated with a one-stage graft urethroplasty following Asopa’s technique, the other seven were treated with a one-stage dartos fascia flap. The success rate of the single-stage graft urethroplasty was 84% (16 of 19 patients). They excluded patients with lichen sclerosus or failed hypospadias repair. Ashraf et al. [25] published a paper on Asopa’s technique with BMG for strictures caused by balanitis xerotica obliterans in boys with a median age of 13 years at the time of the urethroplasty. The median follow-up was 34 months with a success rate of 100%.

The different studies on Asopa’s technique are all heterogeneous in terms of stricture location. Cases in which the meatus and the glandular urethra are involved in the stenosis are very often excluded from analysis. The same goes for strictures caused by lichen sclerosus and failed hypospadias repair(s).

Our study of 40 men undergoing a single-stage dorsal inlay oral mucosal graft urethroplasty according to the Asopa technique for meatal strictures is therefore unique in its kind and cannot be compared to other studies. Furthermore, concerning the substitution material, here is general consensus that if free extragenital tissue is needed, oral mucosal grafts are the standard for augmentation urethroplasty [3]. These can be harvested from the cheek, lip or tongue [6,7,26]. Augmentation urethroplasties with oral mucosal grafts have been widely described and have shown patency rates reaching up to 86% at other stricture sites than the urethral meatus [4,27,28,29]. The oral mucosa has the advantage of being accustomed to a wet environment, is constantly available and has good immunological properties (resistant to infection). It has a thick epithelium, a high content of elastic fibers with a slim lamina propria and an excellent vascularization which makes it an excellent graft with resistance to skin diseases [30], such as lichen sclerosus. Some drawbacks come from the harvesting site with complications such as numbness, salivatory changes and difficult mouth opening. Fortunately these oral discomforts are the majority of the time temporary and minimally disabling [27,28,29].

Simonato et al. [4] evaluated the use of a lingual mucosal graft in distal urethral strictures and found that the mucosa of the tongue is a safe and effective graft material for augmentation urethroplasty. The success rate for penile stricture involving the meatus after failed hypospadias repair using LMG was 67%. A systematic review [29] comparing LMG with BMG showed comparable results concerning urethral patency without a significant difference in overall long-term complications. Surprisingly, in our study the LMG urethroplasties had a significantly higher recurrence rate after 5 years (35% vs. 4% for BMG, *p* = 0.03), although these patients were significantly more treatment naive (respectively 12% and 53%, *p* = 0.006). One hypothesis might be the fact that the lingual mucosa is less thick and thus less resistant to air contact and more likely to atrophy, although they seem to have the same tissue characteristics due to the same embryologic origin.

Our study showed a 5-y RFS of 63% for patients with a failed hypospadias repair versus 90% for non-FHR patients. This difference was not statistically significant but a tendency was observed. This means that FHR patients with recurrence of urethral stricture are more difficult to treat with poorer results due to previous operations and reconstructions. The local tissues have already been used resulting in poor quality. Very often the glans of hypospadias patients is less developed. In these cases, a two-stage urethroplasty is preferable, but a single-stage dorsal inlay augmentation urethroplasty is possible if the patient is keen on avoiding multiple operations.

Unfortunately, due to its observational retrospective design, data on the sexual, erectile and ejaculatory function was too sparse and therefore not analyzed.

With the Asopa technique used to treat meatal strictures one can assume that the skin incision on the ventral side of the distal penis will give the patient some loss of sensibility in that region. Moreover the sagittal incision of the spongiosal body may affect the glans resulting in some floppiness, cold sensation during erection or orgasmic disturbances. On the other hand, given the wildly described collateral vascularization between the cavernosal bodies and the glans, we expect that the impact of this procedure, which is only limited to the distal part of the penis, will be minimal to the overall sexual, erectile and ejaculatory functioning.

We would like to highlight that our population is heterogeneous and that patients with LS or FHR might already have sensitivity problems of their glans. Daneshvar et al. [19] are the first to report patient-reported outcomes following distal urethral reconstruction. Their population was equally heterogeneous. They found no statistical difference in sexual function pre- and postoperatively following urethroplasty (sexual health was evaluated through the ‘Sexual Health Inventory for Men’ score).

As in every study, ours has its limitations. Due to the rare nature of this etiology and the specific technique used, our sample contained only 40 cases. Furthermore, its retrospective character could be prone to intrinsic bias. Another point to mention would be the lack of data regarding sexual function of these patients pre- and postoperatively, which was mentioned in the discussion earlier and which we expect the impact to be minimal. Nevertheless further studies are needed to prove this hypothesis.

## 5. Conclusions

Our study showed that dorsal inlay oral mucosal graft urethroplasty is a safe and feasible technique for selected patients with meatal stenosis. Prospective studies with other potential treatments of meatal stenosis, as well as patient-reported outcomes, are needed to define its exact place in the management for urethral reconstruction.

## Figures and Tables

**Table 1:** Surgical characteristics of 40 patients who underwent single-stage dorsal inlay oral mucosal graft urethroplasty for meatal strictures between 2000 and 2021 stratified by substitute tissue, Lichen Sclerosus and Failed Hypospadias Repair.

	Overall	BMG	LMG	*p*-Value	LS	non-LS	*p*-Value	FHR	non-FHR	*p*-Value
Number of patients; N (%)	40 (100)	25 (62.5)	15 (37.5)	-	18 (45)	22 (55)	-	8 (20)	32 (80)	-
Age at surgery (years); median (IQR)	46 (35–54)	48 (41–54)	35 (29–54)	0.16	48 (41–56)	45 (31–51)	0.18	35 (26–45)	49 (38–54)	0.046
Surgical characteristics										
Length (cm); median (IQR)	2 (1.5–3)	2 (1.5–2.5)	2 (1.25–3)	0.49	2 (1–3)	2 (1.5–3)	0.64	2.5 (2–3.25)	2 (1.25–3)	0.14
Graft width (cm); median (IQR)	2 (2–2.5)	2.45 (2–2.5)	2 (2–2.25)	0.32	2.5 (2–2.5)	2 (2–2.5)	0.20	2 (2–2.5)	2 (2–2.5)	0.96
Follow-up (months); median (IQR)	85 (69–110)	85 (66–126)	90 (74–109)	0.89	97 (47–111)	78 (72–103)	0.70	85 (74–106)	85 (64–111)	0.96
Previous interventions; N (%)										
None	11 (28)	3 (12)	8 (53)	0.006	5 (28)	6 (27)	0.27	4 (50)	7 (22)	0.19
Endoluminal treatment only	7 (18)	7 (28)	0 (0)	-	5 (28)	2 (9)	-	0 (0)	7 (22)	-
Urethroplasty (±endoluminal treatment)	22 (55)	15 (60)	7 (47)	-	8 (44)	14 (64)	-	4 (50)	18 (56)	-

BMG buccal mucosal graft, LMG lingual mucosal graft, LS Lichen Sclerosus, FHR failed hypospadias repair, IQR interquartile range.

**Table 2:** Outcomes of Asopa’s technique for meatal strictures.

	Overall	BMG	LMG	*p*-Value	LS	non-LS	*p*-Value	FHR	non-FHR	*p*-Value
Number of patients; N (%)	40 (100)	25 (62.5)	15 (37.5)	-	18 (45)	22 (55)	-	8 (20)	32 (80)	-
Recurrence	7 (17.5%)	23 (92%)	10 (67%)	0.08	15 (83%)	18 (82%)	1	5 (63%)	28 (88%)	0.13
Need for reintervention	4 (10%)	1 (4%)	3 (20%)	-	2 (11%)	2 (9%)	-	2(25%)	2 (6%)	-
No need for reintervention	3 (7.5%)	1 (4%)	2 (13%)	-	1 (6%)	2 (9%)	-	1 (13%)	2 (6%)	-
5-y RFS, median (SD)	85 (±6)	96 (±4)	65 (±13)	0.03	89 (±7)	81 (±9)	0.85	63 (±17)	90 (±5)	0.08
Complications										
None	29 (72.5%)	18 (72%)	11 (73%)	0.85	16 (89%)	13 (59%)	0.22	5 (63%)	24 (75%)	0.35
G1	6 (15%)	3 (12%)	3 (20%)		1 (6%)	5 (23%)		1 (13%)	5 (16%)	
G2	4 (10%)	3 (12%)	1 (7%)		1 (6%)	3 (14%)		1 (13%)	3 (9%)	
G3a	1 (2.5%)	1 (4%)	0 (0%)		0 (0%)	1 (5%)		1 (13%)	0 (0%)	
Hospital stay (days); median (IQR)	2 (1–2)	2 (1–2)	2 (1–2)	0.66	2 (1–2)	2 (1–2)	0.60	2 (2–2)	2 (1–2)	0.42
Operation time (minutes); median (IQR)	80 (65–90)	80 (66–87)	80 (65–93)	0.62	71 (62–83)	85 (75–91)	0.048	78 (63–100)	80 (65–89)	0.88

SD standard deviation, IQR interquartile range, RFS recurrence free survival, BMG buccal mucosal graft, LMG lingual mucosal graft, LS Lichen Sclerosus, FHR failed hypospadias repair.

**Table 3:** Voiding outcomes of Asopa’s technique for meatal strictures.

	Preop	Postop	*p*-Value
Qmax (mL/s; mean ± SD)	8.36 ± 4.64	21.34 ± 10.77	<0.001
Qav (mL/s; mean ± SD)	6.03 ± 3.43	14.55 ± 7.22	<0.001
VV (mL; mean ± SD)	228 ± 187	341 ± 205	0.017
PVR (mL; mean ± SD)	63 ± 111	36 ± 53	0.353

SD standard deviation, Qmax maximum flow rate, Qav average flow rate, VV voided volume, PVR post void residue.

## Data Availability

The data presented in this study are available on request from the corresponding author.

## References

[1] Mundy A.R., Andrich D.E. (2011). Urethral strictures. BJU Int..

[2] Lumen N., Hoebeke P., Willemsen P., De Troyer B., Pieters R., Oosterlinck W. (2009). Etiology of Urethral Stricture Disease in the 21st Century. J. Urol..

[3] Lumen N., Campos-Juanatey F., Greenwell T., Martins F.E., Osman N.I., Riechardt S., Waterloos M., Barratt R., Chan G., Esperto F. (2021). European Association of Urology Guidelines on Urethral Stricture Disease (Part 1): Management of Male Urethral Stricture Disease. Eur. Urol..

[4] Simonato A., Gregori A., Ambruosi C., Venzano F., Varca V., Romagnoli A., Carmignani G. (2008). Lingual Mucosal Graft Urethroplasty for Anterior Urethral Reconstruction. Eur. Urol..

[5] Palminteri E., Brandes S.B., Djordjevic M. (2012). Urethral reconstruction in lichen sclerosus. Curr. Opin. Urol..

[6] Patterson J.M., Chapple C.R. (2008). Surgical techniques in substitution urethroplasty using buccal mucosa for the treatment of anterior urethral strictures. Eur. Urol..

[7] Horiguchi A. (2017). Substitution urethroplasty using oral mucosa graft for male anterior urethral stricture disease: Current topics and reviews. Int. J. Urol..

[8] Kulkarni S., Barbagli G., Kirpekar D., Mirri F., Lazzeri M. (2009). Lichen Sclerosus of the Male Genitalia and Urethra: Surgical Options and Results in a Multicenter International Experience with 215 Patients. Eur. Urol..

[9] Barbagli G., De Stefani S., Annino F., De Carne C., Bianchi G. (2008). Muscle- and Nerve-sparing Bulbar Urethroplasty: A New Technique. Eur. Urol..

[10] Barbagli G., Selli C., Tosto A., Palminteri E. (1996). Dorsal free graft urethroplasty. J. Urol..

[11] Asopa H.S., Garg M., Singhal G.G., Singh L., Asopa J., Nischal A. (2001). Dorsal free graft urethroplasty for urethral stricture by ventral sagittal urethrotomy approach. Urology.

[12] Malone P. (2004). A new technique for meatal stenosis in patients with lichen sclerosus. J. Urol..

[13] Johanson B. (1953). [The reconstruction in stenosis of the male urethra]. Z. Urol..

[14] Orandi A. (1968). One-stage urethroplasty. Br. J. Urol..

[15] Devine P.C., Horton C.E., Devine C.J.S., Devine C.J.J., Crawford H.H., Adamson J.E. (1963). Use of full thickness skin grafts in repair of urethral strictures. J. Urol..

[16] Venn S.N., Mundy A.R. (1998). Urethroplasty for balanitis xerotica obliterans. Br. J. Urol..

[17] Depasquale I., Park A.J., Bracka A. (2000). The treatment of balanitis xerotica obliterans. BJU Int..

[18] Nikolavsky D., Abouelleil M., Daneshvar M. (2016). Transurethral ventral buccal mucosa graft inlay urethroplasty for reconstruction of fossa navicularis and distal urethral strictures: Surgical technique and preliminary results. Int. Urol. Nephrol..

[19] Daneshvar M., Simhan J., Blakely S., Angulo J.C., Lucas J., Hunter C., Chee J., Alvarado D.L., Perez E.A.R., Madala A. (2020). Transurethral ventral buccal mucosa graft inlay for treatment of distal urethral strictures: International multi-institutional experience. World J. Urol..

[20] Zumstein V., Dahlem R., Kluth L.A., Rosenbaum C.M., Maurer V., Bahassan O., Engel O., Fisch M., Vetterlein M.W. (2020). A critical outcome analysis of Asopa single-stage dorsal inlay substitution urethroplasty for penile urethral stricture. World J. Urol..

[21] Barbagli G., Morgia G., Lazzeri M. (2008). Retrospective outcome analysis of one-stage penile urethroplasty using a flap or graft in a homogeneous series of patients. BJU Int..

[22] Pisapati V.L.N.M., Paturi S., Bethu S., Jada S., Chilumu R., Devraj R., Reddy B., Sriramoju V. (2009). Dorsal Buccal Mucosal Graft Urethroplasty for Anterior Urethral Stricture by Asopa Technique. Eur. Urol..

[23] Pahwa M., Gupta S., Pahwa M., Jain B.D.K., Gupta M. (2013). A comparative study of dorsal buccal mucosa graft substitution urethroplasty by dorsal urethrotomy approach versus ventral sagittal urethrotomy approach. Adv. Urol..

[24] Aldaqadossi H., El Gamal S., El-Nadey M., El Gamal O., Radwan M., Gaber M. (2014). Dorsal onlay (Barbagli technique) versus dorsal inlay (Asopa technique) buccal mucosal graft urethroplasty for anterior urethral stricture: A prospective randomized study. Int. J. Urol..

[25] Ashraf J., Turner A., Subramaniam R. (2018). Single-stage urethroplasty with buccal mucosal inlay graft for stricture caused by balanitis xerotica obliterans in boys: Outcomes in the medium term. J. Pediatr. Urol..

[26] Mangera A., Patterson J.M., Chapple C.R. (2011). A systematic review of graft augmentation urethroplasty techniques for the treatment of anterior urethral strictures. Eur. Urol..

[27] Kumar A., Das S.K., Trivedi S., Dwivedi U.S., Singh P.B. (2010). Substitution urethroplasty for anterior urethral strictures: Buccal versus lingual mucosal graft. Urol. Int..

[28] Sharma A.K., Chandrashekar R., Keshavamurthy R., Nelvigi G.G., Kamath A.J., Sharma S., Venkatesh G.K. (2013). Lingual versus buccal mucosa graft urethroplasty for anterior urethral stricture: A prospective comparative analysis. Int. J. Urol..

[29] Abrate A., Gregori A., Simonato A. (2019). Lingual mucosal graft urethroplasty 12 years later: Systematic review and meta-analysis. Asian J. Urol..

[30] Djordjevic M.L. (2014). Graft surgery in extensive urethral stricture disease. Curr. Urol. Rep..

